# Buffalo Academy of Medicine

**Published:** 1902-07

**Authors:** F. W. McGuire

**Affiliations:** Secretary


### SOCIETY PROCEEDINGS.

### Buffalo Academy of Medicine.

*Stated Meeting, May 20, 1902.*

Reported by F. W. McGUIRE, M. D., Secretary.

MEETING called to order by the president, Dr. Charles G. Stockton, at 8.40 p.m.

The minutes of the last stated meeting, held March 4, 1902, were read and approved.

The council recommended the election of Dr. Eugene Wasdin, U. S. Marine Hospital Service, for non-resident membership and Dr. Edward D. Gibson for resident membership. They were separately balloted for and elected fellows of the Academy.

It was moved and seconded that Section 2 of the by-laws, relating to the annual meeting, be temporarily suspended. Carried.

It was moved and seconded that the annual meeting of the Buffalo Academy of Medicine be held June 17, 1902. Carried.

On motion the commitee appointed to investigate St. Mary's Orphan Asylum and Maternity Hospital was requested to report June 17. Carried.

Election of officers for section of pathology resulted as follows: chairman, Dr. Irving P. Lyon; secretary, Dr. Jacob S. Otto.

A memorial to Dr. Edward Mott Moore, of Rochester, was read by Dr. J. W. Grosvenor, as follows:

IN MEMORIAM—EDWARD MOTT MOORE, M. D.

Dr. Edward Mott Moore, born in Rahway, N.J., on July 15, 1814, completed his earthly pilgrimage of 88 years and passed into his eternal rest at Rochester, on March 3, 1902. His career was marked by high resolves and noble deeds.

We recognise in him a striking illustration of the utility of a thorough preparation as a factor for the highest success in the medical profession. Born of a race of professional teachers he inherited a love for study and investigation. Through classical and scientific courses of instruction his preparation for the study of medicine was unusually thorough. By practical experience in a physician's office, attendance at two of the foremost medical colleges of the country, being graduated from the medical department of the University of Pennsylvania, and by work in the capacity of resident physician of hospitals he became fully equipped for the beginning of professional life.

Taking up his residence in Rochester, in the year 1840 the three score years of his medical career were spent in that city. Those years were devoted to the intense, every-day work of practical medicine and surgery, in both of which branches he gained a wide reputation.

The experiments in the early part of his professional life upon the action of the heart are well known. In after years he gave much professional attention to the treatment of fractures and dislocations. Moore's treatment of Colles's fracture, which differed greatly from the established usage of that day, was the outcome of an investigation of the anatomical conditions which accompany that injury. The results of his investigations and experiments were presented to medical societies and published in medical periodicals. For the Surgical Encyclopedia he wrote an article on fractures and dislocations. His articles bore the stamp of practical information and suggestion.

For the twenty-five years previous to 1883 he occupied the professorial chair of surgery in the Medical College of Buffalo. His connection with this college and his surgical professorship in the medical school at Woodstock, Vermont, Berkshire Medical College of Massachusetts, and Starling Medical College at Columbus, Ohio, gave him a service of forty years as a teacher in which with rare thoroughness he trained many hundreds of medical students and physicians for their future work.

As a lecturer he was clear, concise and convincing. He indulged in no irrelevant platitudes; he clung tenaciously to the subject under consideration. When his lecture, either didactic or clinical, was finished, the hearer felt that he had listened to a speaker who was actuated by a sincere and lofty purpose. His large and well proportioned physique gave him a dignified and exalted appearance in the professorial chair and added to the force of his descriptive language.

He may have been most widely known through his surgical achievements, but his work extended far beyond the domain of surgery; he was a successful practitioner of general medicine and was specially skilful as a diagnostician.

His medical brethren, in recognition of his attainments, placed him in honorable positions in medical societies to which he belonged. He was president of the American Medical Association, of the Medical Society of the State of New York, and American Surgical Association, and delegate to the International Congress of Physicians, held at Copenhagen in 1894.

He was not merely an eminent surgeon and physician, but was a citizen of the broadest type. In Rochester, the city of his adoption, where he lived and labored for more than sixty years, he was an ardent advocate of progress, governmental, commercial, educational, sanitary, social, ethical. He was the only president of the Rochester Park Board and retained that position to the end of his life. Rochester calls him the father of its park system and recognises its beautiful park as his monument. He was also president of the Red Cross Society of Rochester and of the Board of Trustees of Rochester University. He held the rare attitude of being president of all societies of which he became a member.

Dr. Moore was interested not merely in the advancement of his own city, but his desires reached out towards the betterment of mankind everywhere. He was a humanitarian in the largest meaning of that word.

He aimed to become a master in all his work and to this end, by thorough investigation, sounded the depths of whatever problem he undertook to solve. He had lofty ideals and his hand reached out very far toward their attainment. He attained not only to an American but to a world-wide reputation.

In strength of character and broad attainments we may liken him to the gigantic oak, towering above the trees which surround it, spreading its branches far and wide, affording protection from the summer's heat, defying the wintry blasts, growing into the life of centuries. Many a year will pass ere we look upon his like again. It should be ours to emulate and preserve green in memory his many and exalted virtues.

By the citizens of Rochester he will long be remembered for his work in the elevation of the material, intellectual and social conditions of that city. He has been aptly called Rochester's "foremost citizen."

By posterity he will be recognised as standing in the front rank of the American physicians and surgeons of his time.

We congratulate the family, kindred and friends of Edward Mott Moore, M. D., on their high privilege of enjoying for more than fourscore years the rare friendship of a personality of such broad aims and successful work, and tender to them our sincerest sympathy for the great loss they have sustained in his removal from his earthly sphere of labor and companionship.

J. W. Grosvenor,
Eugene A. Smith,
Herman E. Hayd,
*Committee.*

Buffalo, N. Y., May 20, 1902.

On motion the memorial was received and ordered spread upon the minutes and a copy sent to the family.

The Section of Pathology provided the following program:

Dysentery and its causes, Simon Flexner, Professor of Pathology, University of Pennsylvania. Notes on a few cases of smallpox from the Buffalo epidemic of 1901-1902 (stereopticon), David E. Wheeler.

Dr. Flexner's paper was discussed by Drs. Herbert U. Williams, DeLancey Rochester, E. E. Blaauw, Grover W. Wende, Irving P. Lyon, Charles G. Stockton and M. Hartwig.

Dr. Wheeler's paper was discussed by Drs. Grover W. Wende, M. Hartwig and Charles G. Stockton.

Dr. E. E. Blaauw presented a specimen of sarcoma of the choroid of the eye.

Attendance, 50. Adjourned, 10.30 p.m.

